# Post-operative radiological evaluation of a bilateral hypoglossal nerve stimulation system

**DOI:** 10.3389/fsurg.2026.1818247

**Published:** 2026-08-25

**Authors:** A. Kilgue, H.-B. Gehl, N. Abolmaali, L.-U. Scholtz, C. Scherl, I. Todt

**Affiliations:** 1 Department of Otolaryngology, Head and Neck Surgery, Faculty of Medicine OWL, Bielefeld University, Klinikum Bielefeld Mitte, Bielefeld, Germany; 2 Institute for Diagnostic and Interventional Radiology, Klinikum Bielefeld, Bielefeld, Germany; 3 Department of Otolaryngology, Head and Neck Surgery Alexianer St. Gertrauden-Krankenhaus, Berlin, Germany

**Keywords:** computed tomography, hypoglossal nerve stimulation, obstructive sleep apnea, radiological artifacts, x-ray imaging

## Abstract

**Introduction:**

Hypoglossus stimulation implants are a second-line option for the treatment of obstructive sleep apnea (OSA). Similar to other implants, postoperative positional evaluation serves as a quality control measure for the surgical procedure and as a reference for future adjustments. Furthermore, the impact of artifacts on the assessment of the adjacent structures might be of clinical importance. This study aimed to evaluate postoperative radiological positional implant control and artifacts of a bilateral hypoglossal nerve stimulation device.

**Material and methods:**

We retrospectively evaluated radiological imaging of 11 patients on the first day after implantation of a bilateral nervus hypoglossus stimulation device. A variety of radiological imaging procedures were utilized: 8 CT scans (in sagittal and axial planes) and 11 x-ray examinations. Radiological imaging was analyzed regarding the position of the implant, anatomy, and artifacts. The age range of patients varied from 59 to 87 years (mean 69.1 years; SD 9.1 years).

**Results:**

Evaluation of x-ray images did not reveal artifacts that affected the diagnostic accuracy of postoperative position control. In contrast, CT images showed artifacts in the coronal, sagittal, and axial planes, making radiological assessment more difficult. We observed a variation of device position without any effect on functionality. Defined paddle symmetry was found in 8 out of 11 cases.

**Conclusion:**

Postoperative radiologic monitoring of a bilateral hypoglossal nerve stimulator is recommended. A variation of device position could be found.

## Introduction

Obstructive sleep apnea (OSA) is a prevalent sleeping disorder characterized by partial or total obstruction of the upper airway due to a decrease in pharyngeal muscle activity during sleep (1). As the first-line treatment, positive airway pressure (PAP), is established for moderate to severe OSA patients. As PAP therapy underlies a low acceptance rate, hypoglossal nerve stimulation (HGNS) has emerged as an effective and equivalent second-line treatment for patients with moderate to severe OSA with poor adherence to positive airway pressure (PAP) therapy (2–5). A breathing-synchronized unilateral hypoglossal nerve stimulation device [uniHGNS, Inspire Medical Systems (United States of America)] received FDA approval in 2014. A bilateral non-breathing-synchronized HGNS (biHGNS) device represents a further approach to OSA treatment. The Genio (Nyxoah SA, Belgium) system received its European CE Mark in 2019 and recently submitted the fourth and final module of its premarket approval application to the FDA. The Genio is used in adult patients to treat moderate to severe OSA who have not tolerated or refused PAP therapy, or in whom this therapy was not effective. The system involves the implantation of a single device that stimulates the hypoglossal nerve bilaterally, which in turn activates the tongue muscles to maintain an open airway during sleep. The system consists of a stimulator that is implanted during a minimally invasive procedure and positioned over the genioglossus muscle with the electrodes aligned bilaterally to both the left and right. The electrodes are aligned with the left and right branches of the hypoglossal nerve. The stimulation of the hypoglossal nerve leads to contraction of the tongue muscle, which keeps the airways open. Postoperative radiological monitoring of intracorporeal implants is part of the clinical standard and has three functions across all implants: (1) Validation of what has been done. (2) Assessment of how it was performed. (3) Establishing a reference value for postoperative complications. In the field of ENT medicine, these principles can be found in plate treatment for midface fractures or the course of cochlear implant treatment. Here, the position of the electrodes is checked postoperatively. Postoperative position control is also recommended in the course of biHGNS. This postoperative assessment is a reference point for possible future stimulation changes. It can be assumed that the anatomical situation and the surgical variability influence the position of the implant. The presence of HGNS implants is assumed to generate radiological artifacts when patients undergo diagnostic imaging. These artifacts primarily affect modalities like magnetic resonance imaging (MRI) and computed tomography (CT), where interference from metallic components of the device may distort the resulting images. Physical effects causing metal artifacts depend on the composition and geometry of metal implants (6). Radiological parameters of data acquisition and image reconstruction must also be taken into account (7). Metal artifacts lead to undesirable distortions like streaks and shadows in the reconstructed images, which can obscure critical anatomical details and make radiological interpretation more difficult (8).

Radiological assessment of a unilateral HGNS device with a focus on therapeutic outcome and device position monitoring has been described recently (9). Therapeutic outcome of biHGNS implants are well-documented, but literature addressing radiological assessment of biHGNS is to date not available. This retrospective study aimed to evaluate the postoperative variability of the submental-located biHGNS implant position by x-ray and to observe CT artifacts.

## Material and methods

Inclusion criteria were (i) age ≥18 years; (ii) postoperative radiological examinations (CT/x-ray) after bilateral HGNS implantation. Data were collected retrospectively from 11 patients who underwent implantation of a bilateral nervus hypoglossus stimulation device (Genio Implant, Nyxoah, Mont-Saint-Guibert, Belgium) between January 2024 and June 2025.

### Patient characteristics

A total of 11 patients underwent _bi_HGNS surgery. Among these patients, 10 patients (91%) were male whilst 1 patient (9%) was female. The age range of patients varied from 59 to 87 years (mean 69.1 years; SD 9.1 years). The preoperative Apnoea-Hypopnoea Index (AHI) ranged from 16.5 to 64 (mean 41.9), indicating severe obstructive sleep apnea in our study group according to the AHI severity classification. The mean Body Mass Index (BMI) was 29.3 kg/m^2^ (25–34 kg/m^2^), indicating overweight to obesity according to BMI categories. All patients (100%) had dental implants. The known eligibility criteria for _bi_HGNS operation were met by all patients.

### An implantable bilateral hypoglossal nerve stimulation system

The implantable stimulator (IS) is a sterile, small, disposable implant that stimulates both left and right branches of the hypoglossal nerve.

The body of the IS consists of a current-receiving antenna, an electrical circuit, and two lateral legs with two paddle electrodes. Materials with tissue contact: Polyp (p-xylylene) polymer—ParyleneC, coating of the implant. Platinum, the electrodes of the implant. MED 4840/4860—silicone rubber.

Physical description: Length (at the upper plate): 27.5 mm. Inner width (between the paddles in parallel position): 24.3 mm. Height: 20.0 mm. Maximum thickness: 3.2 mm. Total volume: 2.2 cm^3^. Total weight: 3 g.

### Surgery

All patients underwent surgery under general anesthesia. The _bi_HGNS is implanted during a minimally invasive surgical procedure through a small incision under the chin. A nerve monitoring-guided selective hypoglossal nerve stimulation was performed using a nerve integrity monitoring system (NIM 3.0; Medtronic) (10). After NIM-guided separation of the protrusion and retrusion nerve fibers, paddle electrodes of the implantable stimulator were positioned on each side, and the implantable stimulator was sutured on both sides of the genioglossus muscle belly. Using an external stimulator intraoperatively, the correct paddle electrode position on the protruding nerve fibres of the genioglossus and tongue movements was visualized. The incision was closed with skin sutures after a satisfactory response was achieved. In all patients, no adverse events were reported during and after implantation. Surgery was performed successfully, and perioperative stimulation tests were sufficient.

### Radiological imaging

We retrospectively analyzed radiological imaging as part of postoperative position control. A combination of x-ray and CT imaging might maximize diagnostic capabilities and provide comprehensive insights into both the mechanical positioning and integrity of the system. While x-rays serve as the initial, general screening tool, CT imaging allows for a more detailed and accurate assessment. To determine the postoperative position of the _bi_HGNS implant, conventional x-ray images in two planes [lateral and anterior-posterior (a.p.) projection] and/or axial CT scans with reformation in three planes (coronal, sagittal, axial) were performed. A variety of radiological imaging procedures were utilized: 8 CT scans and 11 x-ray examinations were conducted. Eight patients (73%) received postoperative x-ray and CT scans, and three patients (27%) received postoperative x-ray control only.

All patients were scanned in the supine position. A multislice computed tomography (CT) system (Aquilion ONE Prism, Canon Medical Systems Corporation, Otawara, Japan) was used for scanning. The single-energy metal artifact reduction algorithm (SEMAR) was applied to reduce metal artifacts from electrodes (11). SEMAR algorithms employ mathematical techniques to identify and model regions affected by metal artifacts.

The following CT scan parameters were applied: maximum tube current, 260 mA; tube voltage, 120 kV.

x-ray examinations were performed on Philips DigitalDiagnost C90. The protocol utilized exposure 4–18 and the required dose area product was 5.73–33.78 cGy cm^2^.

Visual analysis of images was performed using a medical picture archiving and communication system (PACS, DeepUnity 2.0.2.2, Dedalus S.p.A., Italy).

Anatomical features were measured according to a previous study for a unilateral implant device by Schwab et al. (2018) as follows: mandibular length (in mm), distance mandibula plane-to-hyoid (in mm), distance chin to hyoid (in mm), and angle measurement of hyoid bone to stimulation antenna position were measured using DeepUnity PACS both for CT and/or x-ray. Furthermore, the position of the paddle electronics was analyzed in relation to the mandible and hyoid bone, based on previous findings for a unilateral implant device by Steffen et al. (2020).

The implant position was analyzed using digital reference lines as reference points (see Figure 4).

Symmetry of paddle electrode placement was assessed using two independent parameters: (1) horizontal symmetry, defined as the distance from the midline of the stimulation antenna to the upper edge of the paddle electrodes of each side to ensure symmetrical bilateral stimulation of the hypoglossal nerve, and (2) vertical symmetry, defined as the height difference between the upper edges of the right and left paddle electrodes to prevent oblique placement that could lead to different stimulation thresholds. We defined an acceptable horizontal asymmetry threshold of ≤15 mm of horizontal difference midline to paddle electrode in side-by-side comparison and vertical asymmetry threshold of ≤5 mm in height difference of upper edge paddle electrode in side by side comparison.

Artifacts caused by the implant and/or surrounding pre-existing external materials (e.g., dental implants, dental fillings) and/or soft/bone tissue were analyzed. The degree of metal artifacts severity of the _bi_HGNS implant was assessed as follows: mild (good evaluation of surrounding tissue), moderate (acceptable evaluation of surrounding tissue), and strong (limited evaluation of surrounding tissue).

Since this study is the first description of a positional variability of biHGNS a sample size calculation is not adequate.

### Ethical approval

The study is conducted by the guidelines for human studies and adheres to the World Medical Association Declaration of Helsinki. The study was reviewed and positively evaluated by the Ethical Commission of the Wilhelms Universität Münster (IRB) (2024-809 fS; 25.11.2024). All patients provided written consent for their participation in the study.

## Results

No major perioperative or early postoperative surgical complications (e.g., hematoma, infection, device extrusion) occurred that influenced the anatomical positioning of the hardware or impacted the radiological assessment.

**Figure 1:**
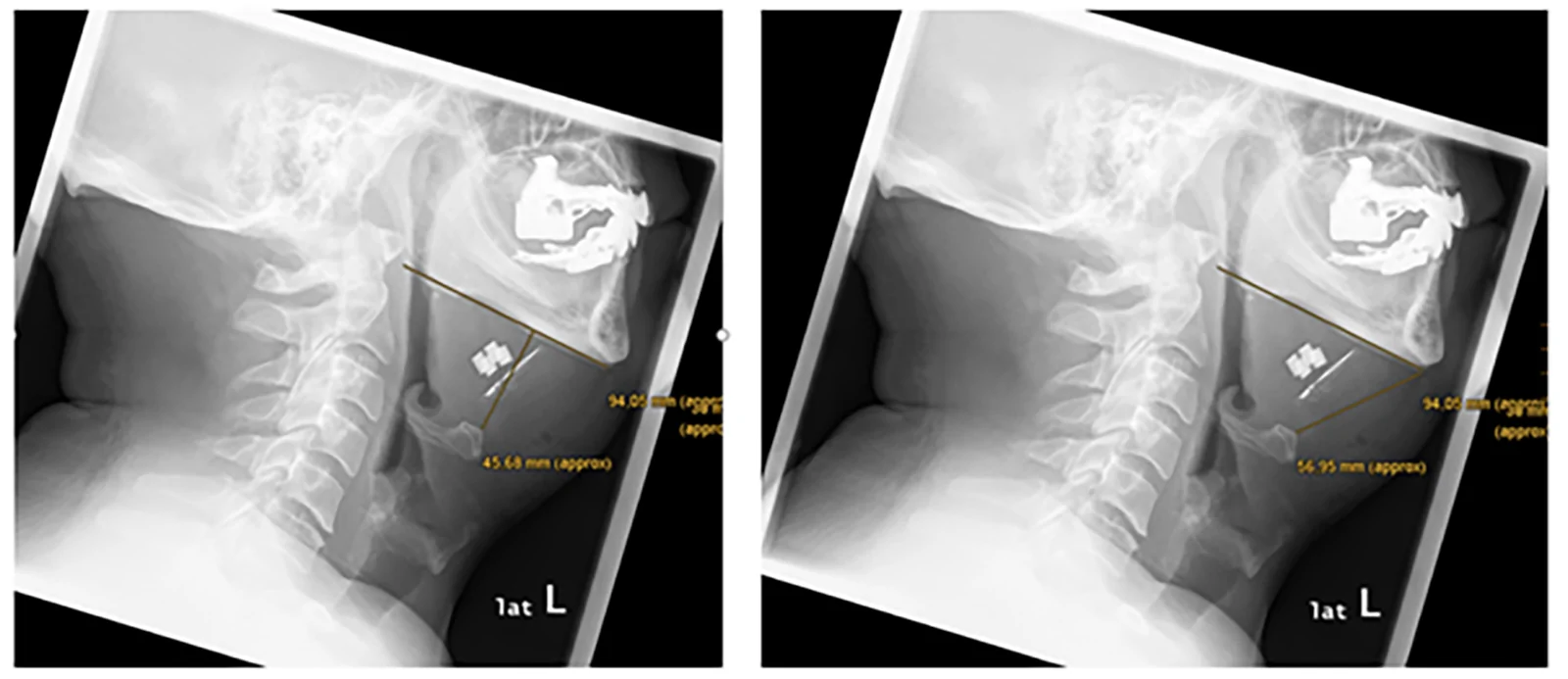
x-ray (lateral). Measurement of mandibular length (both images), distance from mandible plane to hyoid (left image), and distance from chin to hyoid bone (right image).

**Figure 2:**
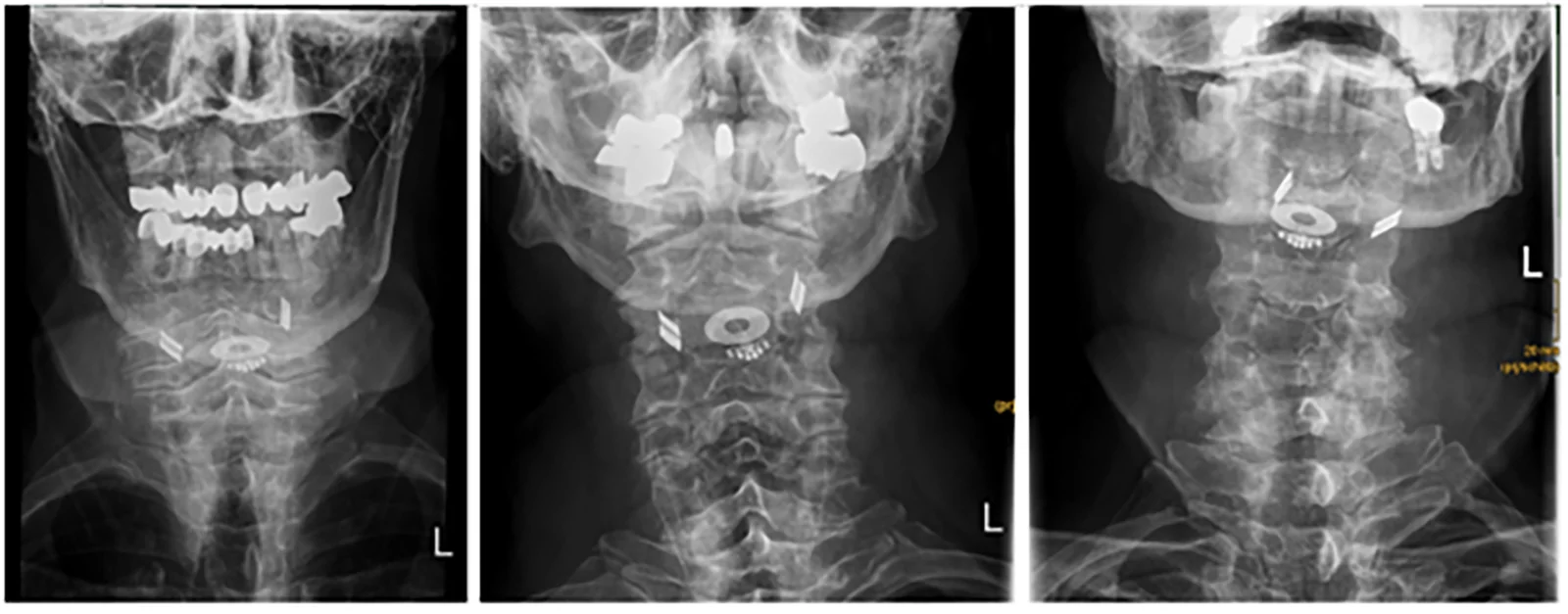
x-ray (a.p.). Asymmetrical alignment of bilateral paddle electrodes in 3 patients.

**Figure 3:**
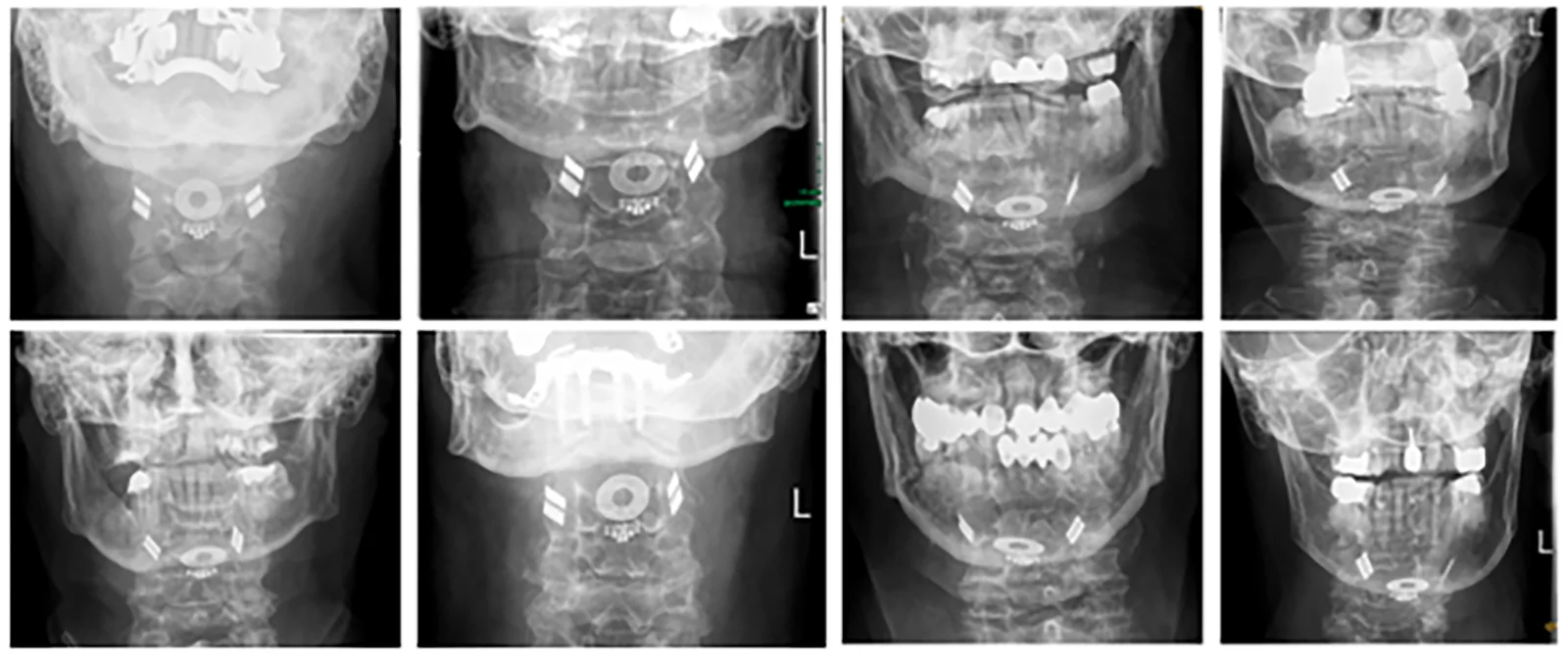
x-ray (a.p.). Symmetrical alignment of bilateral paddle electrodes in 8 patients.

Anatomical features were measured as follows (see Figure 1 and Table 1): mean mandibular length was 102.8 mm (86.0–122.3). Mean distance from chin to hyoid was 60.3 mm (47.1–77.9). Mean distance mandibula plane-to-hyoid was 34.3 mm (20.3–55.3). There was a variability of paddle electrode position. Visual radiological assessment and measurements of the paddle electrode position on the left and right sides revealed symmetrical positions in 8 out of 11 cases (73%) and asymmetrical positions in 3 out of 11 cases (27%) (see Table 1, Figures 2, 3).

**Table 1:** Patient-specific results.

Patient	Mandibular length (in mm)	Distance mandibula plane-to-hyoid (in mm)	Distance chin to hyoid (in mm)	Mean distance midline to paddle electrodes (in mm)	1_distance midline to right paddle electrode (in mm)	2_distance midline to left paddle electrode (in mm)	Horizontal difference midline to paddle electrode right vs. left side (in mm)	Vertical height difference upper edge paddle electrode right vs. left side (in mm)	Alignement paddle electrode symmetry/asymmetry	Angle hyoid-stimulation antenna (in°)	Electrode position proximal/distal to mandible	Electrode position proximal/distal to hyoid
1	87.3	28.7	56.4	21.5	27.7	15.3	12.5	12.2	asymmetry	64.9	proximal	proximal
2	94.1	45.7	57.0	26.4	26.5	26.3	0.2	1.7	symmetry	70.4	proximal	proximal
3	109.3	32.6	48.4	23.7	21.1	26.4	5.3	3.8	symmetry	60.3	proximal	proximal
4	113.9	55.3	76.2	24.2	27.8	20.6	7.2	13.5	asymmetry	73.8	proximal	proximal
5	93.2	23.9	54.5	25.5	26.8	24.2	2.6	2.6	symmetry	35.3	proximal	proximal
6	86.0	20.3	47.1	25.0	24.7	25.3	0.6	3.9	symmetry	19.0	proximal	distal
7	102.0	36.7	64.3	20.3	25.9	14.7	11.2	4.4	symmetry	40.7	proximal	distal
8	122.3	32.1	77.9	18.9	4.3	33.4	29.1	13.5	asymmetry	39.9	proximal	distal
9	110.5	40.3	69.4	25.9	30.4	21.4	9.0	5.0	symmetry	63.5	proximal	proximal
10	117.7	36.2	63.0	26.1	25.6	26.5	0.9	0.6	symmetry	50.0	proximal	distal
11	94.3	25.4	49.3	21.4	21.0	21.8	0.8	3.2	symmetry	39.5	proximal	proximal

**Figure 4:**
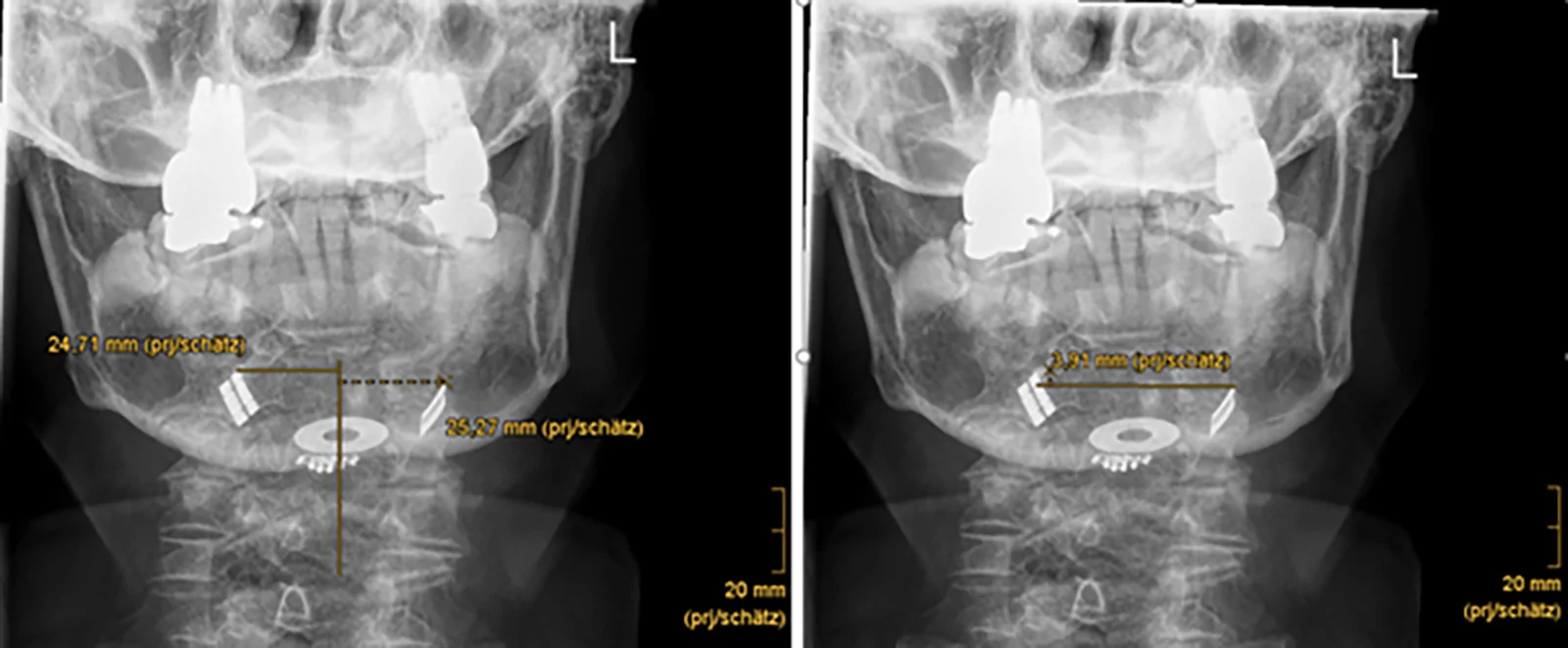
x-ray (a.p.). Quantitative analysis of implant position. Patient with symmetrical horizontal electrode position and vertical symmetrical electrode position.

The average distance (in millimeters) from the midline to paddle electrodes across 11 patients was measured to assess implantation consistency (see Figure 4). Mean distance from midline to paddle electrode was 23.5 mm (18.9–26.4). Mean distance from midline to left respectively right paddle electrode was 23.3 mm and 23.8 mm indicating consistent placement with a moderate horizontal electrode position variation.

**Figure 5:**
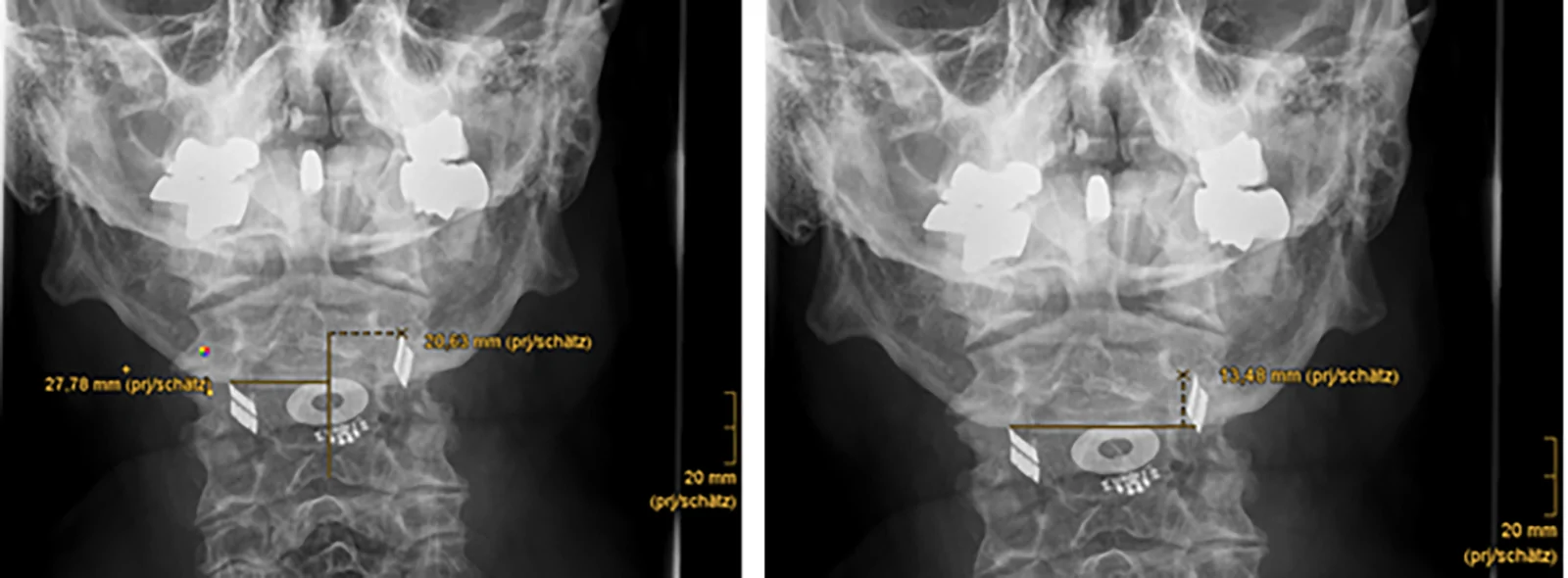
x-ray (a.p.). Quantitative analysis of implant position. Patient with symmetrical horizontal electrode position and vertical asymmetrical electrode position.

Analyzation of vertical height difference between the upper edges of right and left paddle electrodes revealed in 8 out of 11 cases (73%) a symmetrical vertical electrode postion with a vertical asymmetry threshold of ≤5 mm in height difference (see Figure 4). In 3 cases (27%) asymmetry thresholds were exceeded with vertical height differencesof 12.2–13.5 mm (see Figure 5).

**Figure 6:**
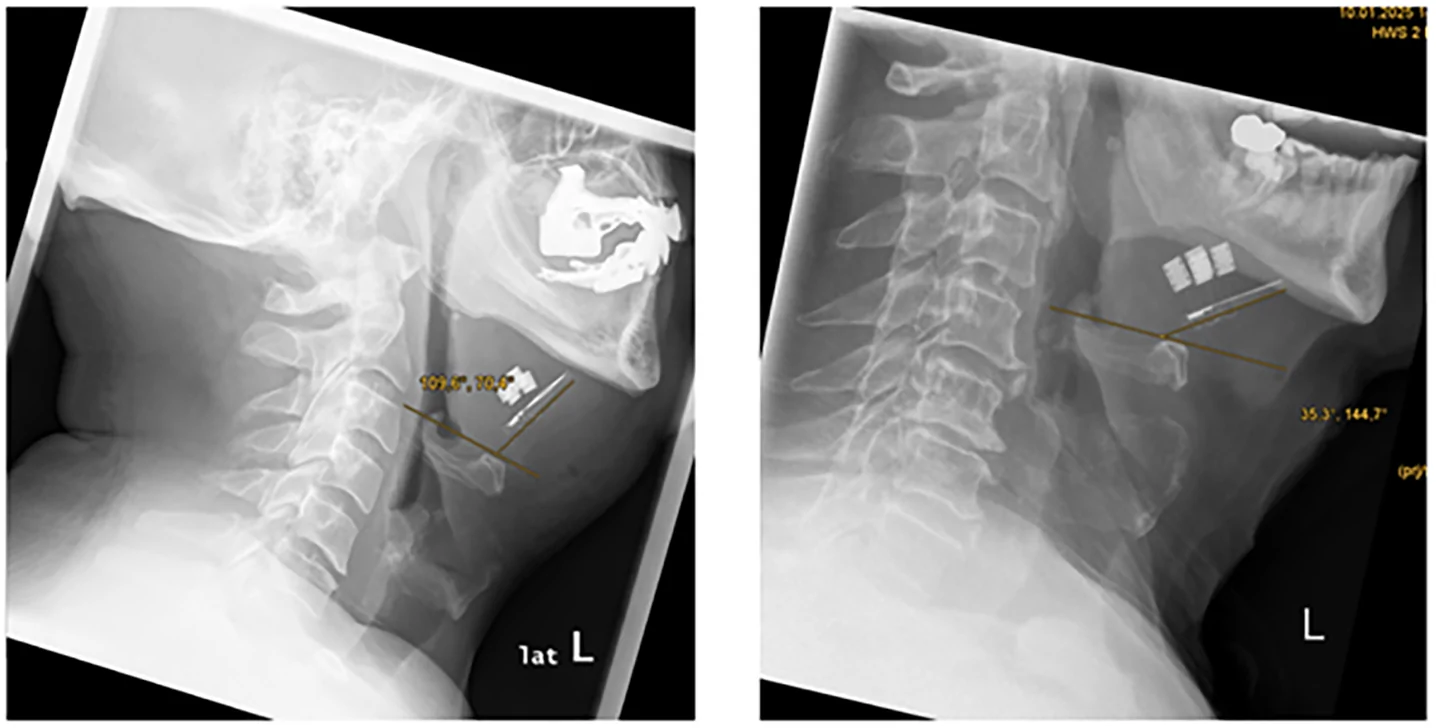
x-ray (lateral). Position of paddle electronics and angle of hyoid to implant (in°).

Evaluation of the hyoid bone to stimulation antenna angle (in°) revealed a mean angle of 50.7° (19.0°–73.8°) (see Figure 6).

**Figure 7:**
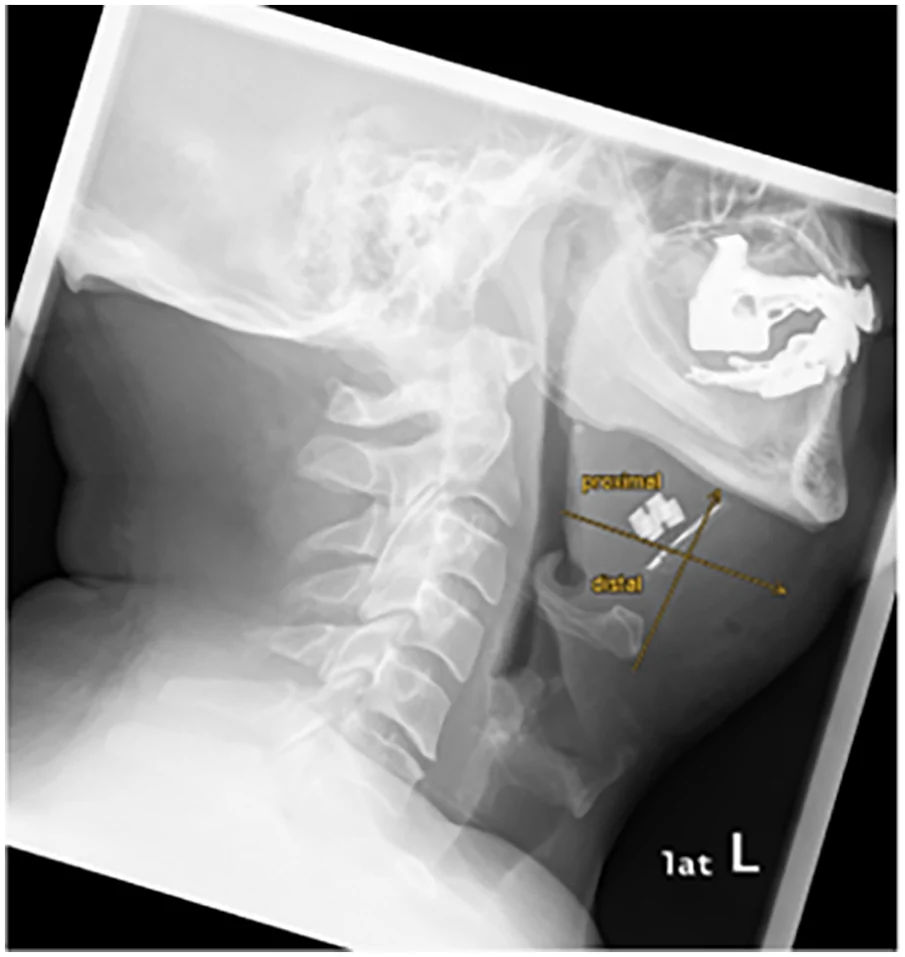
x-ray (lateral). Position of paddle electronics in relation to the mandible.

The paddle electrode positioning was located proximal to the mandible in 11 out of 11 cases (100%) (see Figure 7).

**Figure 8:**
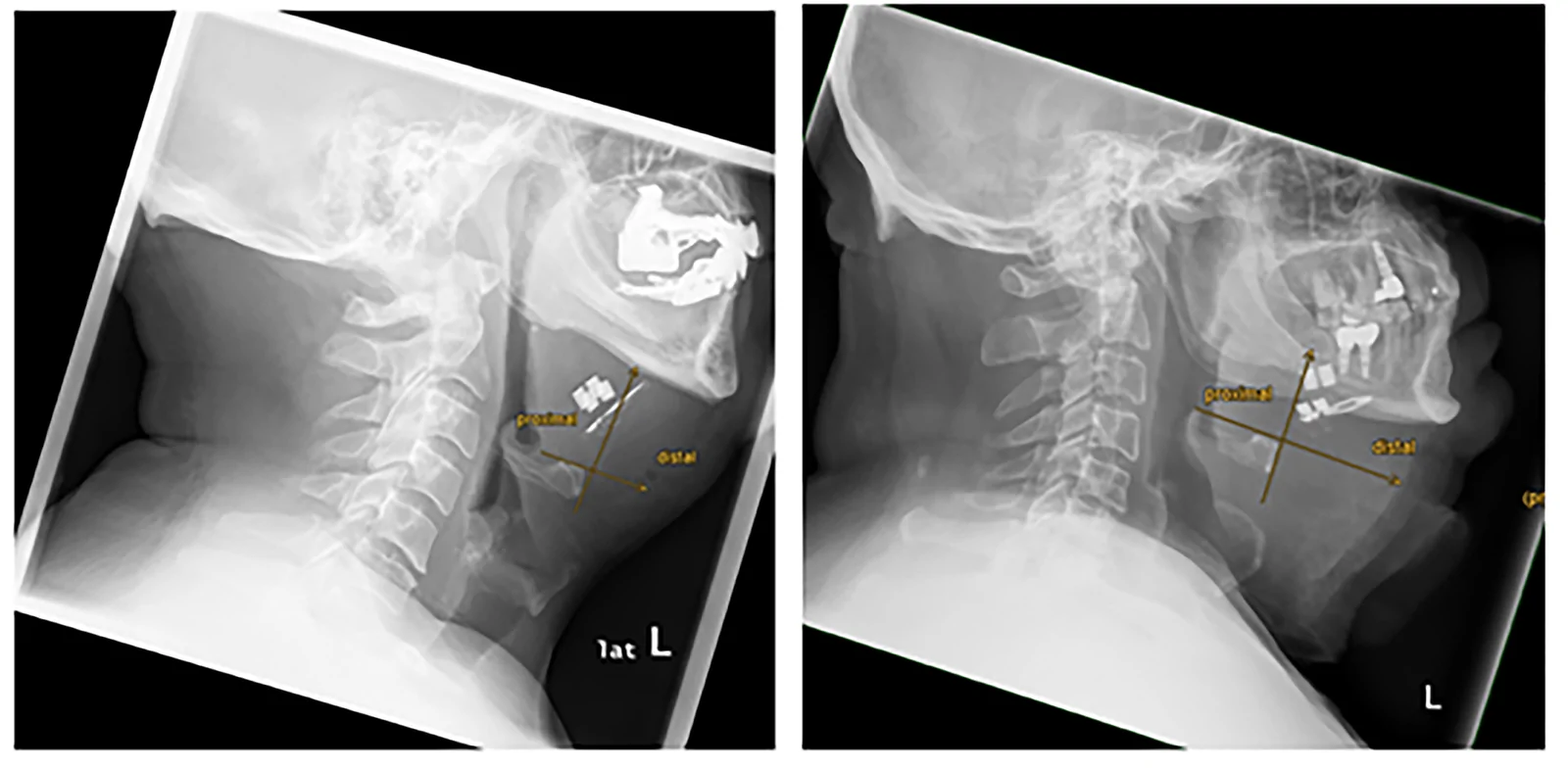
x-ray (lateral). Position of paddle electronics in relation to the anterior margin of the hyoid bone.

The paddle electrode positioning, relative to the anterior margin of the hyoid bone, was proximal in 7 out of 11 cases (64%) and distal in 4 out of 11cases (26%) (see Figure 8).

Evaluation of 11 x-ray images did not reveal artifacts in the lateral and a.p. views that might affect the diagnostic accuracy of postoperative position control. In all 11 cases (100%), images were free of distortions.

**Figure 9:**
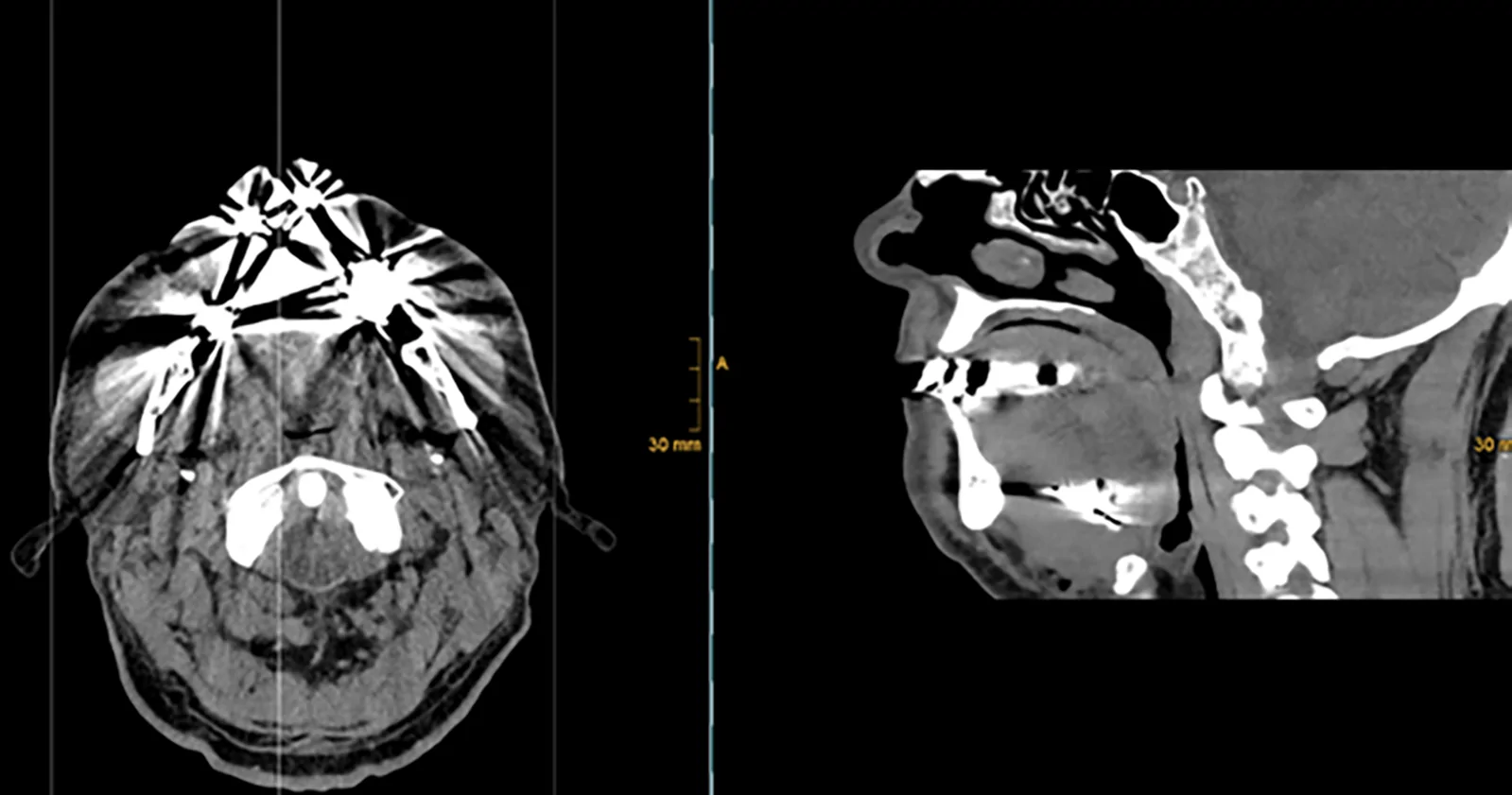
CT (axial and sagittal planes). biHGNS implant position and strong artifacts.

**Figure 10:**
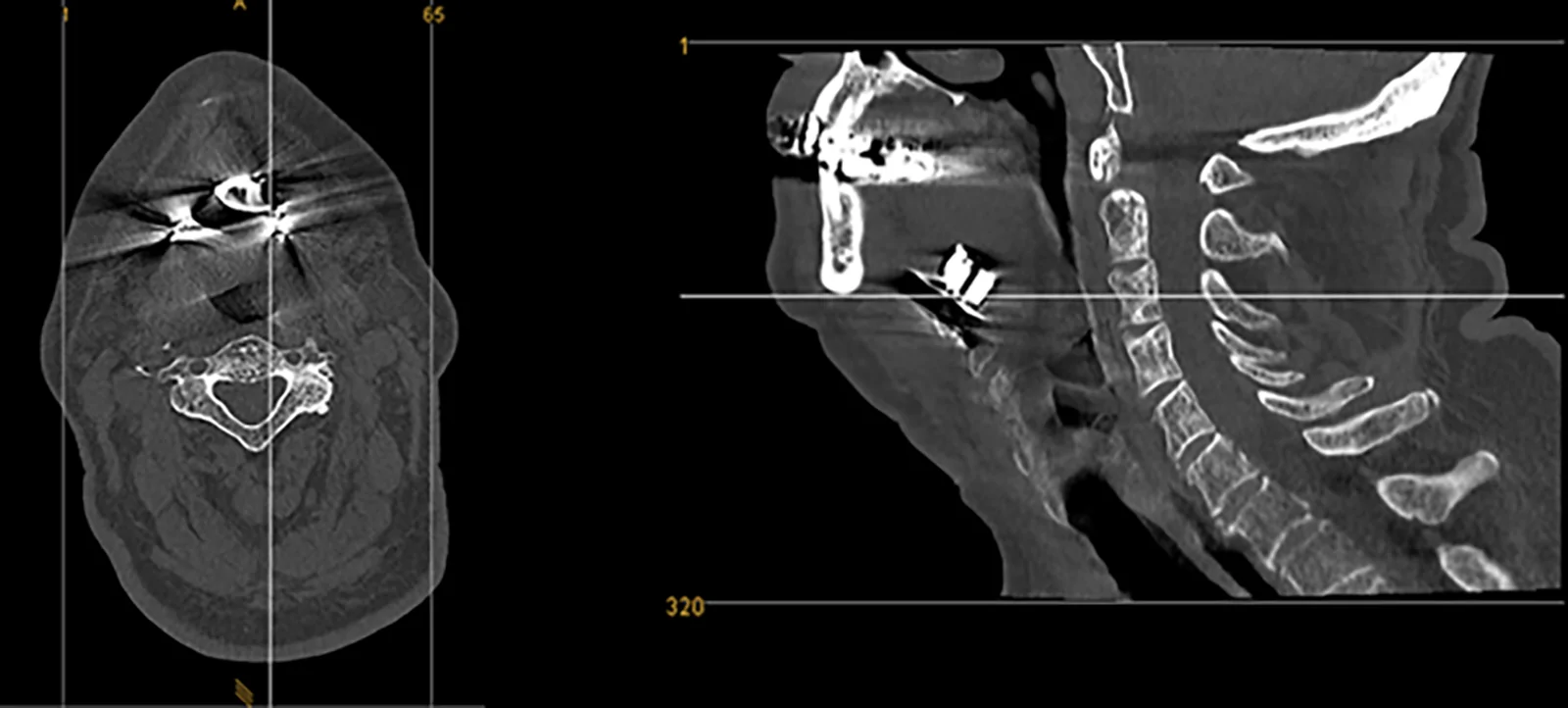
CT (axial and sagittal planes). biHGNS implant position and moderate artifacts.

CT images showed artifacts in all 8 cases (100%). Here, a visual artifact graduation was applied according to recognized artifact intensity (see Figures 9, 10).

Five out of eight cases (63%) exhibited moderate artifacts, with an acceptable evaluation of the surrounding tissue. Three out of eight cases (37%) presented with strong artifacts, which limited the review of surrounding tissue and resulted in significant limitations in postoperative CT image assessment.

In all cases, a regular protrusion of the tongue was observed both intraoperatively and postoperatively, regardless of radiological electrode position.

## Discussion

Postoperative radiological positional control is the standard of care for many implants used in the otolaryngological field (e.g., cochlear implant, plates, etc.). For _bi_HGNS, it acts as a reference point in cases of a complex postoperative adjustment process or malfunction caused by a dislocation.

In case of postoperative hematoma, seroma, or wound infections, radiological monitoring of the implant position can deliver essential insights into the submental region. Due to the age and secondary diseases of patients with obstructive sleep apnea, the detection and characterization of tumors, including primary tumors of the head and neck region, is of particular interest.

Despite the application of a single-energy metal artifact reduction algorithm (SEMAR) to reduce artifacts, CT-based examinations are still affected by artifacts caused by dental implants or metal components of the implantable hypoglossal nerve stimulator. SEMAR cannot eliminate all artifacts, particularly if the metal object is located in a challenging area, such as the floor of the mouth, with anatomical relationships to bones, soft tissue, and dental fillings.

We used bone structures as landmarks for controlling the radiological implant position. We identified variable anatomical features (mandibular length, distance from the mandibular plane to the hyoid bone, distance from the chin to the hyoid bone) based on individual anatomical differences. Furthermore, we analyzed both horizontal and vertical measurements to define the symmetry of paddle placement.

Position control of paddle electronics symmetry was evaluated based on the assumption that an asymmetrical electrode position leads to insufficient nerve stimulation and tongue movement. We observed a variability of implant position without any effect on the functionality of _bi_HGNS.

Contrary to the hypothesis that a proximal electrode position is unfavourable and that an electrode position closer to the mandible reduces the risk of retraction fiber stimulation, no direct conclusions could be drawn about the postoperative stimulation of the nervus hypoglossus in our study group. These results align with those of Steffen et al. (2020). Radiological imaging can therefore be used to determine whether the electrode is in a proximal or distal position. Due to the individual and complex anatomy of the nerve, it is not possible to make a reliable statement postoperatively as to whether one of the desired tongue movements can be expected.

In the present study, postoperative assessment of x-ray images did not reveal artifacts in the lateral and a.p. views that might affect the diagnostic evaluation. x-rays provide 2D images that are more challenging to interpret in cases of complex diagnostic requirements involving soft tissue. It can be difficult to distinguish structures that overlap on an x-ray image, which can lead to misinterpretations. Although the radiation dose is relatively low compared to CT scans, there is still radiation exposure.

Typically, CT scans are more advanced than x-rays in identifying detailed and complex conditions. x-rays provide high-resolution images, but CT scans enable cross-sectional images of the body delivering more detailed views of bones and soft tissue than x-rays. But artifacts caused by metal parts are a fairly common problem in CT imaging. CT images of our study group showed artifacts of varying intensity, ranging from moderate to strong, in all cases, which affected the visual assessment of the previously mentioned anatomical region. CT scans revealed artifacts, which made radiological assessment more challenging. Artifacts only occurred in the recording image plane. In reformatting, metal is denser than soft tissue, but the artifacts cannot leave the recording image plane. Artifacts in CT scans are manageable but require CT scans reformation in at least two planes Ito ensure optimal assessment of the submental region, the mouth base, and the base of the tongue.

Nevertheless, radiological CT assessment is restricted after _bi_HGNS implantation due to artifacts influenced by factors such as individual anatomy measures, previous implants (e.g., dental implants, dental fillings), and the position of the _bi_HGNS implant device. However, the patient's exposure to radiation must be weighed up against the potential gain in knowledge. Besides the disadvantage of higher radiation exposure, CT imaging creates higher costs. A postoperative x-ray can nevertheless be recommended to document the implant position and rule out implant displacement. But knowledge about the number of artifacts is of importance since an overlapping group of patients with _bi_HGNS and head and neck cancer can be assumed in the future.

We recommend x-ray as the standard tool for routine postoperative assessment due to low radiation, low cost, rapid availability, and excellent visualization of the overall hardware geometry. CT imaging Should not be used routinely due to high radiation and significant scatter artifacts from the bilateral components. Instead, CT provides substantial additional value and is strictly recommended in complicated clinical situations, such as (1) suspected surgical site infections or hematomas where the assessment of soft tissue is of critical importance, (2) unexpected postoperative neurological deficits (e.g., severe weakness of the hypoglossal nerve), in order to rule out direct mechanical compression by the anchoring components, (3) ambiguous x-ray findings in which a 3D reconstruction is necessary despite the presence of artifacts.

A limitation of the study is that due to the descriptive study design of a case series, no confirmatory analysis exists. Due to the small number of cases, only exploratory descriptive analyses and no other complex models are possible. As such, no formal sample size calculation has been done and we only report on descriptive findings in terms of mean (standard deviation), numbers and proportions. Consequently, it is not possible to make strong practical implications. While identifying radiological features associated with treatment failure is a vital next step for the field, final clinical responder status could not be established at this immediate postoperative stage due to the lengthy therapy titration phase required for nervus hypoglossus stimulation therapy. However, our findings can be considered as first indicators and basis for further studies and research.

## Conclusion

The initial descriptive results indicate that postoperative radiological monitoring after implantation of a bilateral hypoglossal nerve stimulator is important, but this still needs to be verified in prospective studies. A high variation of device projection in x-ray imaging could be found. The variability in implant position observed in our study group does not affect the implant's functionality. Currently, CT artifacts reduce the accuracy of the localization of the device paddles in relation to anatomical structures.

## Data Availability

The original contributions presented in the study are included in the article/Supplementary Material, further inquiries can be directed to the corresponding author.
